# Inflammation and bacteriophages affect DNA inversion states and functionality of the gut microbiota

**DOI:** 10.1016/j.chom.2024.02.003

**Published:** 2024-03-13

**Authors:** Shaqed Carasso, Rawan Zaatry, Haitham Hajjo, Dana Kadosh-Kariti, Nadav Ben-Assa, Rawi Naddaf, Noa Mandelbaum, Sigal Pressman, Yehuda Chowers, Tal Gefen, Kate L. Jeffrey, Juan Jofre, Michael J. Coyne, Laurie E. Comstock, Itai Sharon, Naama Geva-Zatorsky

**Affiliations:** 1 Department of Cell Biology and Cancer Science, Rappaport Faculty of Medicine, Technion – Israel Institute of Technology, Rappaport Technion Integrated Cancer Center (RTICC), Haifa 32000, Israel; 2 Department of Gastroenterology, Rambam Health Care Campus, Haifa 3109601, Israel; 3 Clinical Research Institute, Rambam Health Care Campus, Haifa 3109601, Israel; 4 Rappaport Faculty of Medicine, Technion – Israel Institute of Technology, Haifa 32000, Israel; 5 Moderna, Inc., Cambridge, MA 02139, USA; 6 Center for the Study of Inflammatory Bowel Disease, Division of Gastroenterology, Department of Medicine, Massachusetts General Hospital Research Institute, Boston, MA 02114, USA; 7 Harvard Medical School, Boston, MA 02115, USA; 8 Program in Immunology, Harvard Medical School, Boston, MA 02115, USA; 9 Department of Genetics, Microbiology and Statistics, School of Biology, University of Barcelona, Avda. Diagonal 643 08028, Barcelona, Spain; 10 Duchossois Family Institute and Department of Microbiology, University of Chicago, Chicago, IL, USA; 11 Migal-Galilee Research Institute, P.O. Box 831, Kiryat Shmona 11016, Israel; 12 Faculty of Sciences and Technology, Tel-Hai Academic College, Upper Galilee 1220800, Israel; 13 CIFAR, MaRS Centre, West Tower 661, Suite 505, Toronto, ON M5G 1M1, Canada

**Keywords:** DNA inversions, phase variation, gut microbiome, bacteriophages, inflammatory bowel diseases, Crohn’s disease, ulcerative colitis, *Bacteroides*, immunomodulation, functional plasticity

## Abstract

Reversible genomic DNA inversions control the expression of numerous gut bacterial molecules, but how this impacts disease remains uncertain. By analyzing metagenomic samples from inflammatory bowel disease (IBD) cohorts, we identified multiple invertible regions where a particular orientation correlated with disease. These include the promoter of polysaccharide A (PSA) of *Bacteroides fragilis*, which induces regulatory T cells (Tregs) and ameliorates experimental colitis. The PSA promoter was mostly oriented “OFF” in IBD patients, which correlated with increased *B. fragilis*-associated bacteriophages. Similarly, in mice colonized with a healthy human microbiota and *B. fragilis*, induction of colitis caused a decline of PSA in the “ON” orientation that reversed as inflammation resolved. Monocolonization of mice with *B. fragilis* revealed that bacteriophage infection increased the frequency of PSA in the “OFF” orientation, causing reduced PSA expression and decreased Treg cells. Altogether, we reveal dynamic bacterial phase variations driven by bacteriophages and host inflammation, signifying bacterial functional plasticity during disease.

## Highlights

• Distinct DNA inversion patterns of Bacteroidales in healthy vs. IBD patients
• Inflamed gut induces DNA inversion of the immunomodulatory PSA of *B. fragilis*
• Bacteriophage levels correlate with bacterial DNA inversions in patients
• Isolated bacteriophage affects PSA phase variation and Treg levels

## Graphical abstract

**Figure:**
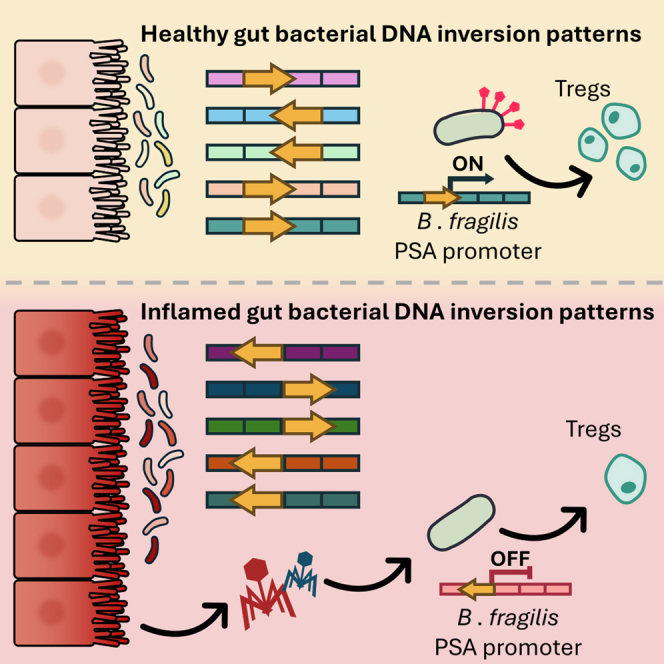

## Teaser

Carasso et al. unveil distinct DNA inversion patterns of Bacteroidales in IBD patients and murine models of gut inflammation. The inflammatory milieu and bacteriophages can reversibly induce the “OFF” state of the anti-inflammatory PSA promoter of *B. fragilis*, offering insights on bacterial functional plasticity during gut inflammation.

## Introduction

Phase variation is the process by which bacteria undergo reversible alterations in specific loci of their genome, resulting in ON-OFF expression of genes.^1^^,^^2^^,^^3^^,^^4^ In Bacteroidales, the dominant order of bacteria in the human gut, phase variation is highly prevalent and is largely mediated by inversions of DNA segments between inverted repeats (IR). These inversions often involve promoter regions dictating transcription initiation of genes or operons functioning as “ON”\”OFF” switches.^5^ In addition, DNA inversions can occur so that new genes are brought from an inactive to a transcriptionally active site by re-orientation or recombination of genomic “shufflons”—mobile genetic elements that facilitate rearrangements, serving as dynamic tools for altering the expressed gene.^6^^,^^7^^,^^8^ Analysis of the orientations of bacterial invertible regions in various host disease states can provide new insights into bacterial adaptation and functional contributions to the disease pathogenesis or its resolution. Phase variation in the gut Bacteroidales often modulates the production of components presented on the bacterial surface^9^ dictating which surface molecules interact, for example, with neighboring microbes (e.g., bacteria and bacteriophages) or with the host.

*Bacteroides fragilis*, a common resident of the human gut, modulates its surface by the phase variable synthesis of its capsular polysaccharides (PS, denoted PSA-PSH). The biosynthesis loci of seven of its eight polysaccharides have invertible promoters that are oriented either “ON” or “OFF” with respect to the downstream PS biosynthesis operon.^5^ Studies have shown that the *B. fragilis* polysaccharide A (PSA) modulates the host immune system by inducing regulatory T cells (Tregs) and secretion of the anti-inflammatory cytokine interleukin (IL)-10.^10^^,^^11^ Moreover, PSA was shown to confer protection against experimental colitis^10^^,^^12^^,^^13^^,^^14^ and thus is regarded as an anti-inflammatory polysaccharide.

Bacteriophages, viruses that specifically target and infect bacteria, can influence the composition of bacterial populations and potentially affect their functionality. Several studies have highlighted the dynamic relationship between Bacteroidales and phages. Campbell et al.^15^ found that infection of *Bacteroides vulgatus* with temperate bacteriophage BV01 results in a repression of bile salt hydrolase activity—changing the bacteria transcriptional profile depending on the integration of the phages into their genome. Porter et al.^8^ and Hryckowian et al.^16^ demonstrated that some capsular PS and other outer surface molecules can affect the susceptibility of *Bacteroides thetaiotaomicron*, to specific phages. Consistent with that, Shkoporov et al.^17^ revealed that phase variation of capsular PS in *Bacteroides intestinalis* influences the infection of CrAss-like phages, establishing a dynamic equilibrium for their prolonged coexistence in the gut. These studies suggest that the interactions between phages and *Bacteroides* may affect the host through changes in bacterial functionality.

Ulcerative colitis (UC) and Crohn’s disease (CD) are multifactorial inflammatory bowel diseases (IBDs), characterized by a compromised mucosal barrier, inappropriate immune activation, and mislocalization of the gut microbiota.^18^^,^^19^^,^^20^^,^^21^ As such, IBDs have emerged as some of the most studied microbiota-linked diseases^22^ and provide an interesting setting for studying gut bacterial functions, their potential effects on the host, and bacteria-phage interactions.

Here, we present an analysis of invertible DNA orientations in the gut microbiota of IBD patients from multiple cohorts and test the hypothesis that inflammation and bacteriophages mediate these differential orientations. Our combined analysis of IBD patient metagenomes and experimental mouse models, focusing on Bacteroidales species, reveals alterations in the orientations of multiple DNA invertible regions during gut inflammation with the potential to modulate the host immune system. Importantly, we show that filtered fecal extracts of IBD patients alter the orientation of the PSA promoter of *B. fragilis*. We show that bacteriophages are correlated with the PSA “OFF” state and that a specific lytic bacteriophage of *B. fragilis* increases the PSA promoter “OFF” population with concurrent decline of host colonic Treg cells. These findings reveal a dynamic interplay between gut inflammation, bacteriophages, and bacterial phase variation, with potential implications for the diagnosis and treatment of IBD.

## Results

### IBD is correlated with quantitative differences in the orientations of multiple Bacteroidales invertible DNA regions compared with healthy controls

**Figure 1:**
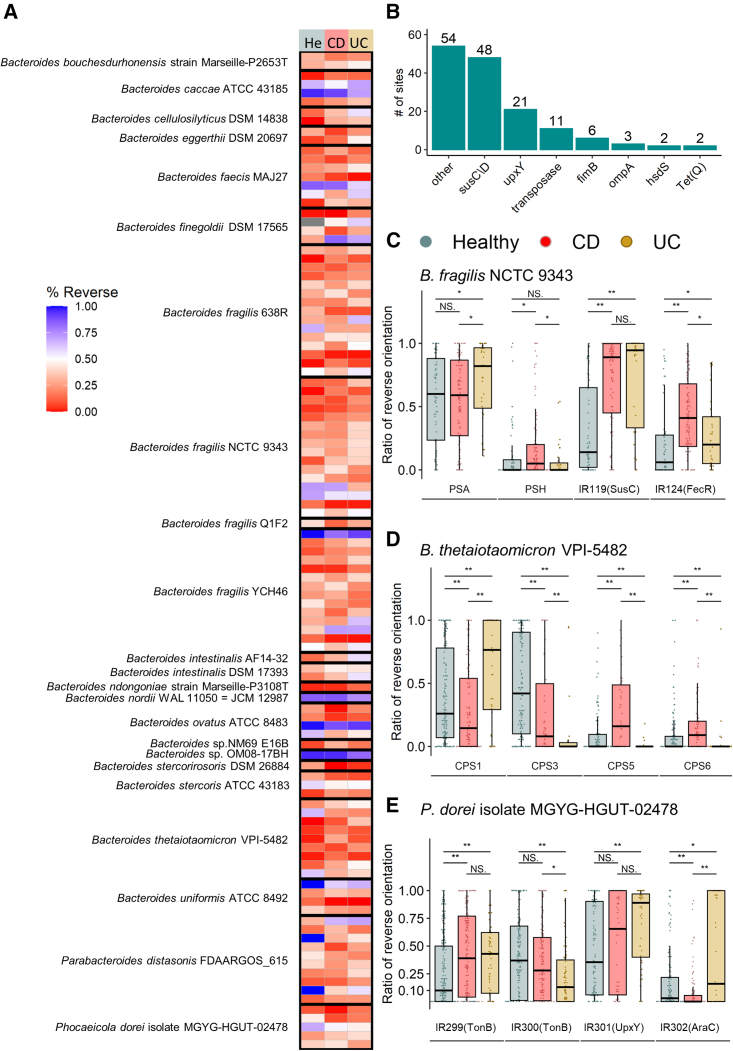
*Bacteroides* species exhibit differential orientations of invertible regions during health and disease (A) Selected significantly differentially oriented invertible DNA regions (inverted repeats (IR) segments, see also Table S1) (Wilcoxon rank-sum test, adjusted p value < 0.05) in at least one comparison between healthy, CD (Crohn’s disease), and UC (ulcerative colitis). Red indicates the forward orientation, and blue represents the reverse orientation in comparison with the reference genome. (B) Prevalence of functional genes in proximity to invertible DNA regions significantly different between healthy individuals and IBD patients. (C) Differentially oriented invertible DNA regions in *B. fragilis* NCTC 9343. PSA, polysaccharide A; PSH, polysaccharide H. (D) Differentially oriented invertible DNA regions in *B. thetaiotaomicron* VPI-5482. CPS, capsular polysaccharide. (E) Differentially oriented invertible DNA regions in *Phocaeicola dorei* MGYG-HGUT-02478. In (C), (D), and (E), data represent the median (line in box), IQR (box), and minimum/maximum (whiskers). (Wilcoxon rank-sum test, ^∗^p < 0.05; ^∗∗^p < 0.01).

As a means to determine if Bacteroidales phase variable molecules may be differentially produced in IBD patients compared with controls, we used the PhaseFinder^9^ software to identify and analyze the relative orientations of DNA inversions in the gut metagenomes of cohorts of IBD patients and control subjects (Table 1). In brief, we first detected putative invertible regions in Bacteroidales genomes by identifying IR and then created a database containing their forward and reverse orientations (compared with the submitted genome sequences). Metagenomic sequences from publicly available datasets were then aligned to the database, resulting in the ratio of the orientations of each invertible region compared with their orientations in the published genome sequence. Our initial analysis of 39 sequenced genomes including 36 human gut Bacteroidales species identified 311 invertible regions. Of these 311 invertible regions, there were 147 that were statistically different in terms of their orientations in IBD patients compared with healthy controls. These included invertible regions from 25 of the reference bacterial genomes (Table S1). These regions included both invertible promoters and intragenic regions of diverse genes, spanning from regulatory genes to those encoding genes for the production of outer surface molecules. Table S1 details the locations of the IR sequences and genes flanking the invertible regions (Figure 1A). The regions in which the orientations differed most significantly between IBD patients and controls were within or in proximity to SusC/SusD-like outer membrane transport systems^23^ and to *upxY* genes, which, in most cases, represent the first gene in the biosynthesis loci of capsular PS (Figure 1B). Four of the five invertible capsular PS promoters of *B. thetaiotaomicron* were differentially oriented between the groups, and three of the seven invertible PS promoters of *B. fragilis* were differentially oriented, including the anti-inflammatory PSA promoter being differentially oriented between healthy and UC patients. The PSA promoter showed a higher percentage (71%) of reverse-oriented reads in IBD patients, compared with 56.2% in the healthy controls (Figure 1C). In the reference genome of *B. fragilis* NCTC 9343, the PSA promoter is in its “ON” orientation, hence, the reverse orientation, found in IBD patients, represents the PSA promoter’s “OFF” orientation. We further identified differential promoter orientations in healthy and IBD cohorts for PULs and PSs promoters of B. thetaiotaomicron VPI-5482 (Figure 1D), and *Phocaeicola dorei*, a bacteria shown to be present in healthy subjects^24^^,^^25^ and correlated with disease activity in UC^26^ (Figure 1E).

**Table 1:** Metagenomic datasets included in this study

Dataset	N (Samples)	CD	UC	Non-IBD	Country	Notes	
IBDMDB	1,283	574	349	360	United States	Longitudinal	Proctor et al.^27^
HMP (3 phases)	320			320	United States		Turnbaugh et al.^28^
MetaHit	122	4	21	97	Denmark and Spain		Qin et al.^29^
1000IBD	331	205	126		the Netherlands		Imhann et al.^30^
GeversD_2014	50	36		14	United States and Canada		Gevers et al. ^31^
LewisJD_2015	303	303			United States and Canada	children with Crohn’s disease, longitudinal, treated	Lewis et al.^32^
Total	2,409	1,122	496	791

### Differential promoter orientation states are induced by inflammation in a dynamic and reversible manner

**Figure 2:**
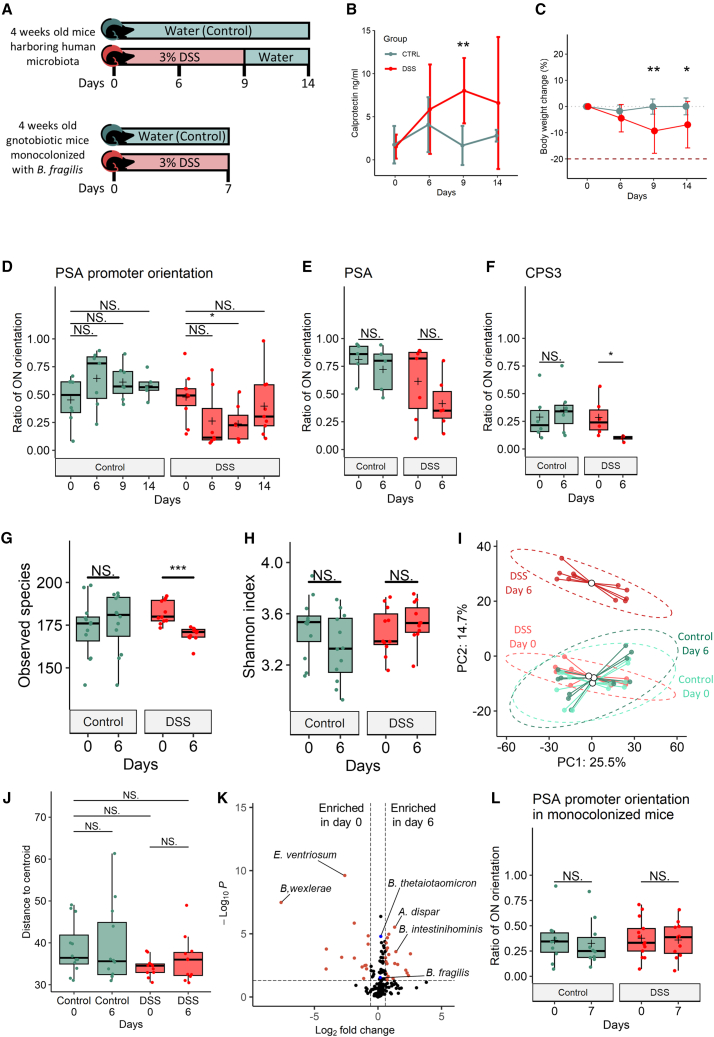
Relative orientation of the PSA promoter of *B. fragilis* is affected by inflammation (A) Illustration of murine model of inflammation. “Humanized” mice harboring human microbiota spiked with *B. fragilis* NCTC 9343, and mice monocolonized with *B. fragilis* NCTC 9343 were exposed to 3% DSS (day = 0 in the illustration) at 4 weeks of age. (B) Calprotectin levels (ng/mL) measured in different days of the experiment. Lines represent the standard deviations. Green: control group; red: DSS-treated mice. (C) The body weight change of mice measured on different days of the experiment. Lines represent the standard deviations. Green: control group; red: DSS-treated mice. (D) Ratio of *B. fragilis* PSA’s promoter “ON” orientation measured by qPCR on different days of the experiment (n = 8–12 in each time point). Data represent the mean (cross), median (line in box), IQR (box), and minimum/maximum (whiskers). (Wilcoxon rank-sum test, ^∗^p < 0.05; ^∗∗^p < 0.01.) Green: control group; red: DSS-treated mice. (E) Ratio of *B. fragilis* PSA’s promoter “ON” orientation measured by the PhaseFinder tool, in different days of the experiment. Data represent the median (line in box), IQR (box), and minimum/maximum (whiskers) (Wilcoxon rank-sum test, ^∗^p < 0.05; ^∗∗^p < 0.01.) Green: control group; red: DSS-treated mice. (F) Ratio of *B. thetaiotaomicron* CPS3’s promoter “ON” orientation measured by the PhaseFinder tool on different days of the experiment. Data represent the median (line in box), IQR (box), and minimum/maximum (whiskers) (Wilcoxon rank-sum test, ^∗^p < 0.05; ^∗∗^p < 0.01.) Green: control group; Red: DSS-treated mice. (G) Alpha-diversity (observed species) between groups and timepoints. Data represent the median (line in box), IQR (box), and minimum/maximum (whiskers) (Wilcoxon rank-sum test, ^∗^p < 0.05; ^∗∗^p < 0.01, ^∗∗∗^p < 0.001.) Green: control group; red: DSS-treated mice. (H) Alpha-diversity (Shannon index) between groups and timepoints. Data represent the median (line in box), IQR (box), and minimum/maximum (whiskers) (Wilcoxon rank-sum test, ^∗^p < 0.05; ^∗∗^p < 0.01, ^∗∗∗^p < 0.001.) Green: control group; red: DSS-treated mice. (I) Beta diversity (Aitchison distance) between groups and time points. Principal coordinates analysis (PCoA) of Aitchison distances between bacterial communities of different groups and time points. Each point represents a single sample, colored according to group and timepoints: light green: control at day 0; green: control at day 6; light red: DSS-treated at day 0; red: DSS-treated at day 6. The mean (centroid) of samples in each group is indicated with a blank circle. Ellipses represent 0.95 confidence intervals of each group. (J) Beta-dispersion values (Aitchison distances from the centroid) between groups and timepoints. Data represent the median (line in box), IQR (box), and minimum/maximum (whiskers) (betadisper, p > 0.05.) (K) Differential bacterial abundance between DSS-treated mice on day 0 and day 6 detected by the Maaslin2 algorithm. Red dots indicate differentially abundant bacteria that were determined by adjusted p value < 0.05 and log_2_ fold change >1 and <−1, respectively. Blue dots indicate *B. fragilis* and *B. thetaiotaomicron*. (L) Ratio of *B. fragilis* PSA’s promoter “ON” orientation measured by qPCR on different days in gnotobiotic mice monocolonized with *B. fragilis* NCTC 9343. Data represent the median (line in box), IQR (box), and minimum/maximum (whiskers) (Wilcoxon rank-sum test, ^∗^p < 0.05; ^∗∗^p < 0.01.) Green: control group; red: DSS-treated mice. See also Figure S1 and Table S2.

To assess the dynamics of the *B. fragilis* PSA promoter orientation under inflammatory conditions, we designed a longitudinal experimental mouse model during which we analyzed the PSA promoter orientation over time. Germ-free (GF) mice were colonized with the gut microbiota from a healthy human microbiota, and subsequently spiked with *B. fragilis* NCTC 9343, termed “humanized” mice. The progeny of these mice were used for the experiments (n = 8–12 mice per experimental group, dextran sodium sulfate [DSS] vs. control). Experimental colitis was induced by adding 3% DSS to the drinking water^33^ for 9 days after which DSS was replaced with water until day 14 (Figure 2A). Inflammation was monitored by the levels of calprotectin in the stool (Figure 2B), a commonly used biomarker for inflammation, and by mouse weight loss (Figure 2C). Stool was collected before, during and after DSS treatment to analyze orientations of specific invertible DNA regions, using quantitative PCR (qPCR) to observe the state of the PSA promoter (Figures S1A–S1C). The orientation of the *B. fragilis* PSA promoter is variable to some extent in both the control and DSS-treated mice and was significantly altered on day 9 in the DSS-treated mice along with the inflammatory state (Figures 2B–2D). At the beginning of the experiment, ∼45% of the population had the promoter in the “ON” orientation (Figure 2D). A significant decline in these percentages was observed six days after DSS was introduced (down to an average of ∼26%) and remained at around ∼24% on average on day nine. By day 14, when the mice were starting to gain weight and the calprotectin levels were decreasing (Figures 2B and 2C), the bacterial population returned to an average of 40% “ON,” similar to the beginning of the experiment, and to the control group, which did not receive DSS (mean of 58% of the *B. fragilis* population “ON”) (Figure 2D).

Since the frequency of *B. fragilis* with the PSA promoter oriented “ON” declined in the majority of the DSS-treated mice (Figure S1D), we performed metagenomic sequencing from stool collected at days 0 and 6 for analysis of orientations of invertible regions in other Bacteroidales species and genomic regions (Table S2).

This analysis validated the decline in the frequency of the “ON” orientation of the *B. fragilis* PSA promoter (Figure 2E), aligned with the qPCR results (Figure S1E), and agreeing with the human metagenomic analysis (Figure 1C). In addition, we identified a decline in the frequency of the “ON” orientation of the CPS3 promoter of *B. thetaiotaomicron* during inflammation in mice (Figure 2F), consistent with the results of the IBD vs. healthy human metagenomic analysis where it also showed lower ratios of the “ON” orientation in IBD patients, equivalent to reverse-oriented reads in comparison with the reference genome (Figure 1D).

The bacterial composition of the gut microbiota in control mice was stable throughout the experiment but varied in the DSS-treated mice. Bacterial richness (alpha-diversity, observed species) declined during inflammatory conditions (Figure 2G), while evenness (alpha-diversity, Shannon index) remained constant (Figure 2H). The beta diversity (Aitchison distance) was significantly altered (Figure 2I), whereas the beta-dispersion (Aitchison distance) was not significantly altered (Figure 2J).

Inflamed mice showed a decrease in relative abundances of *Blautia* species and *Eubacterium ventriosum*, with a concomitant increase in *Barnesiella intestinihominis* and *Alistipes dispar* (Figure 2K; Table S2). The relative abundance of *B. fragilis*, which exhibited differential orientations of invertible DNA regions, was not significantly altered during inflammation (Figure 2K).

To better assess the role of inflammation on the orientation of the *B. fragilis* PSA promoter we repeated the experiment using gnotobiotic mice monocolonized with *B. fragilis* NCTC 9343 (n = 8–12 mice per experimental group, DSS vs. control), excluding influences of altered microbiota (Figures 2A and S1F). The PSA promoter orientation was not altered in the monocolonized mice (Figure 2L), along with the bacterial abundances (Figure S1G) over the course of the experiment (with or without DSS), suggesting a role of the microbiota in driving the “OFF” orientation of the PSA promoter during inflammation.

### The inflammatory milieu of a complex human microbiota results in the PSA promoter “OFF” orientation

**Figure 3:**
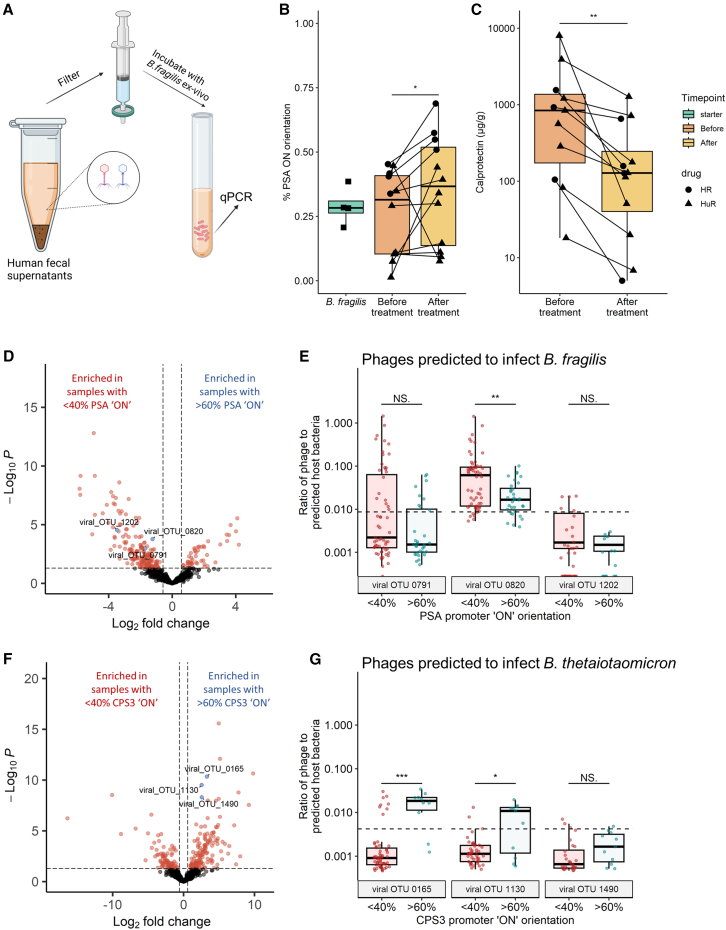
Evidence of phage correlation with *B. fragilis* polysaccharide A promoter orientation (A) Experimental design of culturing *B. fragilis* in patients' fecal filtrates. (B) Ratio of the “ON” orientation of the PSA promoter of *B. fragilis*, measured by qPCR, after *ex vivo* exposure to fecal filtrates of IBD patients before and after treatment with anti-TNF (4 patients on infliximab [HR] and 7 patients on Humira [HuR]). Data represent the median (line in box), IQR (box), and minimum/maximum (whiskers) (one-sided Wilcoxon rank-sum test, ^∗^p < 0.05.) Green: *B. fragilis* grown in a 1:1 ratio of M9 and PBS not exposed to fecal filtrates; orange: *B. fragilis* exposed to fecal filtrates of patients before anti-TNF treatment; yellow: *B. fragilis* exposed to fecal filtrates of patients after anti-TNF treatment. Each dot represents 3 individual experiments; lines connect experiments from the same patient; shapes are determined by the patients’ treatments: circle, HR; triangle, HuR. (C) Calprotectin levels (μg/g) measured in patients’ feces. Data represent the median (line in box), IQR (box), and minimum/maximum (whiskers) (one-sided Wilcoxon rank-sum test, ^∗^p < 0.05.) Dots represent samples; lines connect samples from the same patient; shapes are determined by the patients’ treatments: circle, HR; triangle, HuR. (D) Differential viral taxonomic units’ abundances, (from the IBDMDB cohort, count table from Nishiyama et al.^34^), between samples with low “ON” orientation of the PSA promoter (<40%) and high “ON” orientation (>60%). Differentially abundant viral taxonomic units were detected by the DeSeq2 algorithm (Wald test, p < 0.01). Red dots indicate differentially abundant bacteria that were determined by p value < 0.01 and fold change >1.5 and <−1.5, respectively. (E) Phage-to-host abundances ratios of viral OTUs predicted to infect *B. fragilis* and detected in (D). Data represent the median (line in box), IQR (box), minimum/maximum (whiskers), dots represent individual samples. Dashed line represents the mean phage-to-host ratio of all phages predicted to infect *B. fragilis* in all samples (mean = 0.009). (F) Differential viral taxonomic units' abundances, (from the IBDMDB cohort, count table from Nishiyama et al.^34^), between samples with low “ON” orientation of the CPS3 promoter (<40%) and high “ON” orientation (>60%). Differentially abundant viral taxonomic units were detected by the DeSeq2 algorithm (Wald test, p < 0.01). Red dots indicate differentially abundant bacteria that were determined by p value < 0.01 and fold change >1.5 and <−1.5, respectively. (G) Phage-to-host abundances ratios of viral OTUs predicted to infect *B. thetaiotaomicron* and detected in (F). Data represent the median (line in box), IQR (box), minimum/maximum (whiskers), dots represent individual samples. Dashed line represents the mean phage-to-host ratio of all phages predicted to infect *B. thetaiotaomicron* in all samples (mean = 0.03). See also Figure S2 and Table S3.

We next sought to examine factors of the complex microbiota that result in a decreased percentage of the *B. fragilis* population with the PSA promoter oriented “ON” during inflammation. To do so, we exposed *B. fragilis* NCTC 9343 to fecal filtrates from IBD patients. CD and UC patients were recruited from the Rambam Health Care Campus (RHCC). Fecal samples were collected before and after treatment (Infliximab [HR] or Humira [HuR] therapy, 4 and 7 patients respectively). Both treatments are antibodies targeted against tumor necrosis factor-α (TNF-α), an inflammatory cytokine increased in IBD patients. *B. fragilis* NCTC 9343 was cultured in fecal filtrates to mid-log phase, at which time, DNA was extracted for qPCR analysis of the PSA promoter orientation (Figure 3A). *B. fragilis* exposed to fecal filtrates of patients before treatment showed higher ratios of the population with the PSA promoter oriented “OFF,” while *B. fragilis* exposed to fecal filtrates after treatment showed higher ratios of the PSA promoter oriented “ON” (Figure 3B). This observation was in line with the fecal calprotectin concentrations, which were high before treatment and decreased after treatment, inversely correlating with PSA promoter “ON” orientation (Figure 3C). These results demonstrate that the population of bacteria with the PSA promoter in each orientation varies in the inflamed and non-inflamed gut, with a higher percentage of the population in the “OFF” orientation under inflammatory conditions, and a shift toward the “ON” orientation following reduction in inflammation (Figures 3B and 3C). Particularly, treatment responders had a significant correlation of PSA “ON” with lower calprotectin levels, compared with the non-responders (Figure S2A). This change could be due to induction of inversion under these conditions or to selection of a preferred promoter state.

### Investigating associations of phage with PSA promoter orientation

Fecal filtrates contain a mixture of bacterial and host metabolites, cytokines, antibodies, viruses, bacteriophages, and other components. Bacteriophages were shown to be associated with intestinal inflammation and IBD in several studies,^35^^,^^36^^,^^37^^,^^38^^,^^39^ including Nishiyama et al.^34^ who characterized temperate bacteriophages and their bacterial hosts in the IBDMDB metagenomics database. Here, using phage sequences from the IBDMDB database, we compared the relative abundances of these phages between samples that displayed lower (<40%) or higher (>60%) ratios of the PSA promoter “ON” orientation. Differential relative abundance analysis revealed 108 viral OTUs enriched in patients with lower ratios of the PSA promoter “ON” orientation after false discovery rate (FDR) correction, compared with 24 viral OTUs enriched in patients with the higher ratios of the “ON” orientation (Figure 3D; Table S3). Three of the bacteriophages enriched in the lower ratios of the PSA promoter “ON” orientation were predicted to infect *B. fragilis*^34^—viral OTUs 0791, 0820, and 1202. To assess whether the relative abundances of the bacteriophages were accompanied with lower relative abundances of *B. fragilis*, the phage-to-host abundance ratio was calculated for each phage-*B. fragilis* pair. Two of the OTUs whose relative abundances were correlated to the PSA promoter “OFF” orientation, viral OTUs 0791 and 0820, also showed higher phage-to-host abundance ratios (Figures 3E and S2). Intriguingly, these two viral OTUs were found to be more abundant in active CD and UC.^34^

We conducted the same analysis comparing samples with high (>60%) to low (<40%) “ON” orientation ratios of the CPS3 promoter of *B. thetaiotaomicron*. Although there were differences in the virome compositions between these groups, no *B.-thetaiotaomicron*-associated bacteriophages were correlated with the “OFF” orientation of the CPS3 promoter (i.e., the orientation, which we identified as associated with the disease) (Figure 3F; Table S3). We found three *B. thetaiotaomicron* associated with viral OTUs—0165, 1130, and 1490—slightly associated with the “ON” orientation of the CPS3 promoter (the orientation that we identified as associated with healthy controls) (Figures 3G and S2). In addition, these bacteriophages displayed low phage-to-host ratios (Figure 3G). To note, these OTUs were not previously associated with disease.^34^

### Bacteriophage exposure correlates with *B. fragilis* PSA promoter “OFF” state

Since a higher abundance of bacteriophages was correlated with the PSA promoter in the “OFF” orientation, we sought to study whether encounter with bacteriophage can be associated with altered orientation of the PSA promoter in *B. fragilis*. To this end, we isolated from sewage Barc2635, a *B. fragilis* NCTC 9343 specific lytic bacteriophage (Figure S3A), sequenced its genome, (deposited to GenBank, accession: MN078104), and characterized its morphology by electron microscopy (Figure S3B).

**Figure 4:**
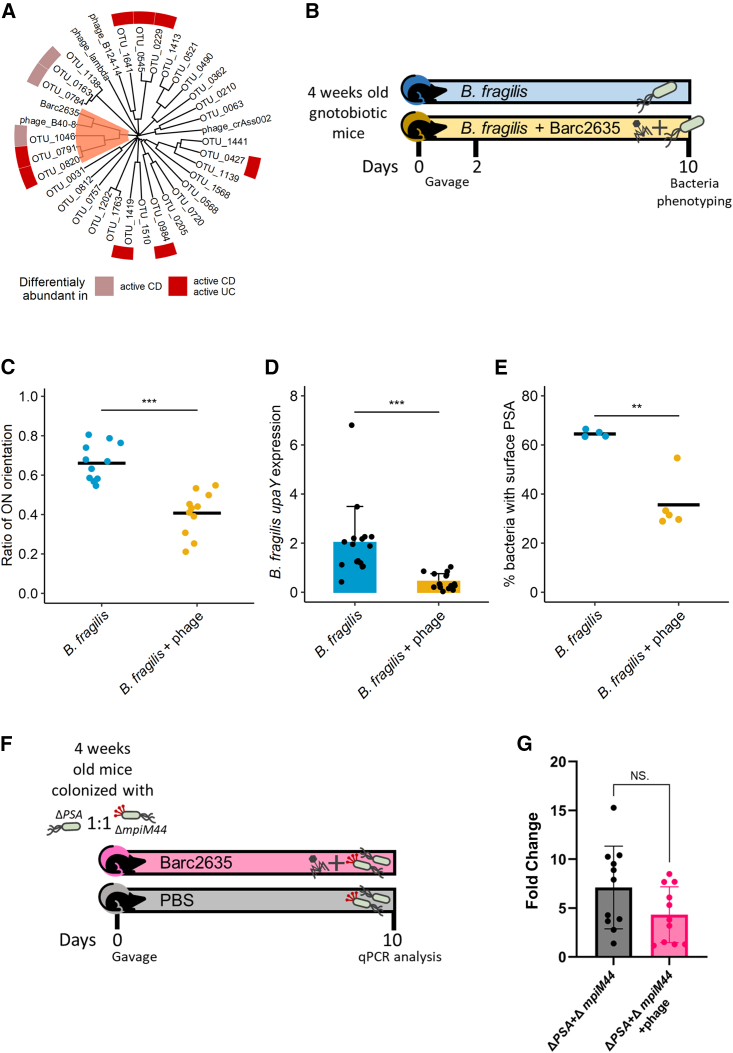
Phage exposure alters the expression of the PSA locus and surface PSA (A) Phylogenetic tree based on the whole genome of viral OTUs identified as bacteriophages against *B. fragilis* as well as *Bacteroides* bacteriophages Barc2635, B40-8, B124-14, crAss002, and *Enterobacteria* phage lambda. MAFFT was used to perform multiple sequence alignment, and the average-linkage method was used to construct the phylogenetic tree. Colors denote the association between the viral OTUs abundances in Nishiyama et al.^34^, IBDMDB cohort, with active Crohn’s disease (light red) or with both active Ulcerative colitis and active Crohn’s disease (red). See also Figure S3. (B) Experimental design of *in vivo* experiments. (C) Ratio of *B. fragilis* PSA’s promoter “ON” orientation measured on day 10 by qPCR in fecal samples of gnotobiotic mice monocolonized with *B. fragilis* with and without the Barc2635 bacteriophage. Horizontal lines represent the means. Blue: control group; yellow: Barc2635-treated mice. Each dot represents a mouse. (Mann-Whitney test, ^∗∗∗^p < 0.001.) (D) Expression levels of *upaY* in ceca of gnotobiotic mice monocolonized with *B. fragilis* with and without the Barc2635 bacteriophage at day 10. Levels are shown as 2ˆ(-ΔCT) with *rpsL* as a reference gene. Each dot represents a mouse, and error bars represent the standard deviations. (Mann-Whitney test, ^∗∗∗∗^p < 0.0001.) (E) PSA presence on the surface of *B. fragilis* exposed to the Barc2635 bacteriophage, detected by anti-PSA antibodies on day 10. Bacteria were analyzed by flow cytometry for the expression of PSA, using Rabbit anti-PSA antibodies. Horizontal lines represent the means. Each dot represents a mouse.| (F) Experimental design of *in vivo* competition experiments. (G) Fold change of Δpsa\ΔmpiM44 at day 10 and day 0 in the Barc2635 bacteriophage treated group (pink) compared with Δpsa\ΔmpiM44 at day 10 and day 0 in the control group (black). Error bars represent the standard deviations. Each dot represents a mouse (Mann-Whitney test, p > 0.05.) See also Figure S4.

Barc2635 is a lytic double-stranded DNA bacteriophage of 45,990 bp with a GC content of 38.9%, containing 67 putative CDS belonging to the tailed phages unified in the class Caudoviricete, formerly *Caudovirales*, (Figure S3C). Interestingly, increased abundance of bacteriophages from this class were found to be correlated with IBD patients (UC and CD).^23^ We analyzed the sequence similarities of Barc2635, with the *B. fragilis*-associated bacteriophages identified in the IBDMDB^34^ as well as from other studies^40^^,^^41^^,^^42^ and found that Barc2635 is most similar to a cluster of bacteriophages, including viral OTUs 0791 and 0820 which were associated with active disease^34^ (Figures 4A and S3D).

To test whether exposure to this bacteriophage leads to changes in relative orientations of the PSA promoter of the *B. fragilis* population, we monocolonized GF mice with *B. fragilis* NCTC 9343 in the presence or absence of Barc2635 and analyzed the PSA promoter DNA inversion state (Figure 4B). In response to bacteriophage, a higher percentage of the population had the PSA promoter in the “OFF” state (Figure 4C). To further analyze the consequence of PSA promoter inversion we measured the mRNA expression of the first gene in the PSA biosynthesis locus, *upaY*,^43^ which requires the PSA promoter to be oriented “ON.” Figure 4D shows a significant reduction in *upaY* expression in mice containing Barc2635 in comparison with mice with bacteria alone. To directly measure the levels of PSA on the surface of the *B. fragilis* population, we used specific antibodies and monitored surface PSA by flow cytometry. The percentage of bacterial cells synthesizing PSA from mice with bacteriophage Barc2635 was significantly lower in comparison with bacterial cells from mice without the bacteriophage (Figure 4E), in agreement with the *upaY* expression levels and the PSA promoter orientation. In the two groups of mice (i.e., *B. fragilis* alone and *B. fragilis* with Barc2635), the CFU levels of *B. fragilis* were different by 0.5 a log (Figure S4A), and no phages were detected in the *B. fragilis* monocolonized group (i.e., without bacteriophage) (Figure S4B).

### Barc2635 infects both ΔPSA and constitutively expressing PSA mutants

To distinguish whether the PSA promoter “OFF” state that resulted from exposure to bacteriophages was due to induction or selection, we studied the interaction of Barc2635 with two mutants of *B. fragilis*: Δ*psa*^44^ and Δ*mpiM44*.^43^ The former mutant lacks the PSA biosynthesis locus, and the latter has the PSA promoter locked in the “ON” orientation, and therefore, constitutively synthesizes PSA. *In vitro* phage infection assays revealed comparable infection efficacy (Figure S4C) and no competitive advantage of either of the mutants during exposure to the bacteriophage (Figures S4D–S4F). In agreement to the *in vitro* results, there was no selectivity of the bacteriophage toward either of the mutants in mice that were initially colonized with equal ratios of the two mutants (Figures 4F and 4G). To note, the Δ*psa* mutant exhibited higher fitness in colonizing GF mice, however, this advantage remained unchanged regardless of exposure to the Barc2635 (Figures S4G and S4H). This was further confirmed by comparing the ratio of Δ*psa/*Δ*mpiM44* on day 10 with the ratio on day 0 between the treated group and the control group (Figure 4G). These data show that mutants lacking PSA do not have a fitness advantage during exposure to Barc2635.

### Bacteriophage-driven phase variation in *B. fragilis* results in a reduction of Treg cells

**Figure 5:**
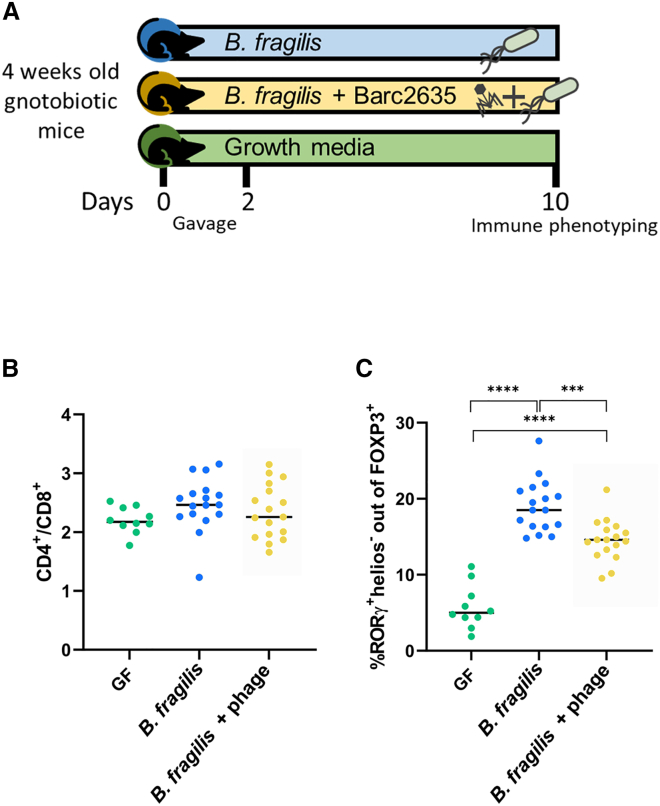
Bacteriophage-driven phase variation in *B. fragilis* results in reduction of Tregs (A) Experimental design of *in vivo* experiments. (B) CD4^+^ to CD8^+^ ratio out of CD45^+^TCR*β*^+^ live cells in colon. Single cells were isolated from colon lamina propria. Immune cells were analyzed by flow cytometry. Horizontal lines represent the means. Each dot represents a mouse. Green, GF mice; blue, mice monocolonized with *B.fragilis;* yellow, mice monocolonized with *B.fragilis* and Barc2635. (C) ROR*γ*t^+^ helios^-^ percentages out of FOXP3^+^ cells in colon. Single cells were isolated from colon lamina propria. Immune cells were analyzed by flow cytometry. Horizontal lines represent the means. Each dot represents a mouse. Green, GF mice; blue, mice monocolonized with *B.fragilis;* yellow, mice monocolonized with *B.fragilis* and Barc2635. ^∗∗∗^p < 0.001, ^∗∗∗∗^p < 0.0001, one-way analysis of variance (ANOVA). See also Figure S5.

It is well established that the PSA of *B. fragilis* NCTC 9343 induces Tregs (CD4^+^Foxp3^+^RORgt^+^) in the colonic lamina propria of mice.^13^^,^^45^^,^^46^^,^^47^ To monitor the effect of Barc2635 during *B. fragilis* colonization to the CD4^+^Foxp3^+^RORgt^+^ Tregs population, we extracted cells from the colonic lamina propria and immunophenotyped them by flow cytometry (Figure 5A). We found that in the presence of Barc2635, the CD4^+^Foxp3^+^RORgt^+^ Tregs population is decreased concomitant with the decrease in *upaY* transcription, coinciding with less surface production of the immunomodulatory PSA (Figures 5B and 5C).

## Discussion

Phase variations, prevalent in host-associated species, especially the gut Bacteroidales,^9^ contribute to bacterial fitness in changing ecosystems. Reversible DNA inversions lead to phase variable synthesis of numerous molecules (e.g., surface, regulatory, and other molecules), and as such, confer functional plasticity. Our study reveals that the phase variable states of certain molecules correlate with gut inflammation, with potential implications on host physiology. By analyzing six different human gut metagenomic datasets with samples from IBD patients and healthy individuals, and using controlled mouse experiments, we identified multiple invertible genomic regions that map to 25 different Bacteroidales strains that are differentially oriented in disease and health. Notably, we find that both intergenic regions (e.g., promoters) and intragenic regions (e.g., genomic “shufflons”) exhibit altered orientations in gut inflammatory conditions—affecting gene expression.

The most prevalent genomic regions with differential orientations during inflammation and health were in PS promoters and near *susC/susD* homologs—both with immunomodulatory potential. SusC-like proteins are abundant β-barrel outer membrane proteins involved in nutrient acquisition in Bacteroidetes. Recently, an epitope of SusC proteins was shown to elicit T cell responses in IBD patients and healthy controls,^48^ suggesting that operons that include SusC homologs might confer immunomodulatory properties to the bacteria. Among the phase variable PSs, the anti-inflammatory PSA promoter of *B. fragilis* showed a higher percentage of reverse-oriented reads in IBD patients compared with healthy controls, indicating that the “OFF” orientation was more prevalent in IBD patients, potentially limiting the protective, anti-inflammatory, effects of PSA. These results align with a previous study^49^ that focused on the PSA promoter of *B. fragilis* in IBD patients using PCR digestion of biopsy samples. We further find that some DNA inversion states, like the PSA promoter of *B. fragilis*, are dynamic and can respond to changes in their local environment, specifically during inflammation.

Intriguingly, the preferential “OFF” orientation of the PSA promoter of *B. fragilis* did not occur to the same extent in inflamed monocolonized mice as it did in inflamed mice colonized with a human microbiota and when bacteria were exposed to patients’ fecal filtrates. These results suggest that environmental factors in inflamed humanized mice and IBD patients are necessary for this effect. Our analysis of phage OTUs in the IBDMDB cohorts revealed enrichment of *B. fragilis*-associated phage OTUs in samples where the PSA promoter was present in the “OFF” orientation. In addition, exposure of *B. fragilis* to an isolated bacteriophage, Barc2635, resulted in a higher frequency of the PSA promoter “OFF” orientation, suggesting a role for gut bacteriophages in bacterial phase variation.

Our analyses were not able to distinguish the cause of the change in DNA orientation during inflammation and phage exposure. It is possible that these conditions induce inversions to the “OFF” orientation but may also result from selection of bacteria that are not expressing PSA. Comparison of mutants that are unable or that constitutively synthesize PSA revealed similar susceptibility to Barc2635 infections, suggesting that the observed decrease in PSA expression may be the result of induction of inversion rather than selection pressures. If so, this area is ripe for future mechanistic understanding.

The anti-inflammatory effects of PSA are mediated by upregulation of induced Tregs (Foxp3+RORgt+) and subsequent induction of IL-10 secretion.^12^^,^^13^ We found that the Tregs populations decreased in *B. fragilis* monocolonized mice infected with Barc2635, suggesting that the increase in bacteria with the PSA promoter in its’ “OFF” orientation has implications on the host immune system, linking bacteriophage exposure to alterations in bacterial functionality with concomitant effects on host physiology. Applying the same analysis to *B. thetaiotaomicron*’s CPS3, we observed bacteriophages that are more abundant in samples with a higher ratio of CPS3 “ON” oriented promoters, however, the bacteriophage to host abundances ratios per sample were too low to conclude that *B. thetaiotaomicron* bacteriophages lead to populations with altered CPS3 synthesis.

Altogether, we demonstrate differential orientations of Bacteroidales invertible regions in human IBD and identify bacteriophages as a potential trigger. These phase variations result in bacterial functional plasticity affecting the host immune system, correlating with inflammation. Bacteriophages are one factor that alters bacterial phase variable states. However, within the inflamed gut, there are numerous factors that may potentially alter the invertible states of Bacteroidales populations. These include the microbiome^34^^,^^36^^,^^50^^,^^51^^,^^52^ (e.g., neighboring microbes), the gut metabolome,^50^ an altered immune system,^53^ and physical alterations in the intestine such as abnormal pH concentrations,^54^ osmotic^55^^,^^56^ and oxidative stress.^57^^,^^58^ For example, Tropini et al.^59^ showed that PEG-induced osmotic perturbation resulted in an increased ratio of CPS4 to CPS5 in *B. thetaiotaomicron*.

This study demonstrates that alterations in invertible genomic regions, and consequently molecular phase variations in gut bacteria during IBD, influence the host immune system and inflammatory state. These phase variations can occur in response to changes in gut environmental factors during inflammation, including, but not limited to bacteriophages. Future studies integrating bacterial DNA inversions and phase variation analyses may illuminate the role of bacterial functional plasticity in additional physiological states, possibly resulting from different environmental factors, which may drive alterations in microbe-host interactions.

### Limitations of the study

The six datasets analyzed in this study included adults and children, healthy controls, and inflamed patients (before and after treatment) and longitudinal samples. The impact of each of these factors, and the impact of diet and lifestyle, merit further research. The PhaseFinder^9^ algorithm that we applied identifies DNA inversions in short reads. As such, our analysis does not include other phase variation mechanisms like duplications or insertions. Also, analysis of short reads of metagenomics sequencing data may overlook inversions resulting from long structural genomic alterations.^3^^,^^7^^,^^60^ Furthermore, metagenomic sequencing data averages the whole bacterial population of samples, implying a dilution factor for low abundant bacteria. Moreover, genomic inversion sites might interfere with genome sequence assemblies, leading to incomplete genomes, and thus could be overlooked when applying tools that search inversions in reference genomes. Also, our bacteriophage computational analysis, which is based on Nishiyama et al.,^34^ might have overlooked some of the bacteriophages in the patients, since it relies on prior analysis of MAGs that were predicted as temperate phages. This study used an identified and isolated lytic bacteriophage for *B. fragilis*, further studies are required to elucidate the functional effects of IBD-associated lytic and temperate bacteriophages. With these limitations notwithstanding, our study highlights the importance of considering bacterial phase variation in the context of IBD and its potential impact on inflammation and on altered microbe-host interactions.

## STAR★Methods

### Key resources table

**Table:** 

REAGENT or RESOURCE	SOURCE	IDENTIFIER
**Antibodies**		

CD45	Biolegend	Cat# 103138
TCR_b	Biolegend	Cat# 109222
CD19	Biolegend	Cat# 115530
TCR_g/d	Biolegend	Cat# 118118
CD4	Biolegend	Cat# 100510
CD8	Biolegend	Cat# 100730
FoxP3	eBioscience	Cat# 17-5773-82
Helios	Biolegend	Cat# 137220
RORgt	eBioscience	Cat# 12-6988-82

**Bacterial and virus strains**		

*Bacteroides fragilis* NCTC 9343	ATCC	ATCC 25285
Bacteriophage Barc2635	This study	N/A
*B. fragilis* lacking mpi gene- *B. fragilis* NCTC 9343 Δ*mpiM44*	Chatzidaki-Livanis et al.^43^	N/A
*B. fragilis* lacking PSA biosynthesis genes - *B. fragilis* NCTC 9343 Δ*PSA*	Coyne et al.^44^	N/A

**Biological samples**		

Fecal samples collected from mice	This study	N/A
Fecal samples collected from IBD patients	Rambam Health Care Campus	N/A

**Chemicals, peptides, and recombinant proteins**		

Dextran sodium sulfate (DSS)	TdB	Cat#: DB001-500 G
DNAse	New england biolabs	Cat#: M0303S
qScript cDNA Synthesis Kit	Quantabio	Cat#: 95049-100-2
SYBR® Green mix	Thermo Fisher Scientific	Cat#: AB-4385614
BRPM broth	Condalab	Cat#: CL-1451
PEG (6,000-12,000 MW, 8%)	Merck	Cat# :25322-68-3
Caesium chloride	Thermo Fisher Scientific	Cat# :7647-17-8
Tris-Cl	Alfa Aesar	Cat# :15424539
MgCl2	Merck	Cat# :7786-30-3
Tris ph7.5	Thermo Fisher Scientific	Cat# :15424539
EDTA	Merck	Cat# :60-00-4
Brain heart infusion (BHIS)	Becton Dickinson	Cat# :237500
NH_4_Cl	Alfa Aesar	Cat# :12125-02-9
Na_2_HPO_4_	Merck	Cat# :7558-79-4
KH_2_PO_4_	Merck	Cat# :7778-77-0
NaCl	Merck	Cat# :7647-14-5
CaCl_2_	Merck	Cat# :10043-52-4
MgSO_4_	Merck	Cat# :7487-88-9
Glucose	Merck	Cat# :50-99-7
L-cysteine	Merck	Cat# :7048-04-6
FeSO_4_	Strem	Cat# :7782-63-0
VitB12	Glentham Life Sciences	Cat# :68-19-9
Vitamin K	Merck	Cat# :84-80-0
Hemin	Alfa Aesar	Cat# :16009-13-5
Agar	Merck	Cat# :9002-18-0
Sodium Azide	Merck	Cat#: 26628-22-8
FBS	Biological industries	Cat# :04-127-1A
PBSx10	Thermo Fisher Scientific	Cat# :02-023-5A
Permeabilization Buffer	Thermo Fisher Scientific	Cat# :00-8333-56
Fixation Buffer	Thermo Fisher Scientific	Cat# :00-8222-49
2% rat serum	Stemcell Technologies	Cat#: 45135
Hoechst dye	Thermo Fisher Scientific	Cat#: 62249

**Critical commercial assays**		

ZymoBIOMICS DNA Miniprep Kit	Zymo	Cat#: ZR-D4300
ZymoBIOMICS RNA Miniprep Kit	Zymo	Cat#: R2001
Qubit™ dsDNA Quantification Assay Kit	ThermoFisher	Cat#: Q32850
IDT for Illumina DNA/RNA UD indexes	Illumina	Cat#: 20027213
Nextera DNA CD indexes	Illumina	Cat#: 20018708
MiSeq Reagent Kit v2	Illumina	Cat#:MS-102-2002
NovaSeq 6000 S4 Reagent Kit v1.5	Illumina	Cat#: 20028312
PhiX Control v3	Illumina	Cat#: FC-110-3001
Millex®-GV Filter Unit (Sterile)	Merck	Cat#: SLGV033RS
GasPak (BBL) jars	Becton Dickinson	Cat#: 260626
LIAISON® Calprotectin	Diasorin	Cat# :318960
Lamina Propria Dissociation Kit, mouse	Miltenyi Biotec	Cat#: 130-097-410

**Deposited data**		

Raw data files of mice feces metagenomic sequencing	This study	SRA: PRJNA916364
Bacteriophage Barc2635 genome	This study	GenBank: MN078104
Human-associated metagenomics assembled genomes used to identify representative Bacteroides species	Pasolli et al.^61^	http://segatalab.cibio.unitn.it/data/Pasolli_et_al.html
Human-associated metagenomics assembled genomes used to identify representative Bacteroides species	Almeida et al.^62^	ENA: ERP108418
Human-Associated metagenomics assembled genomes used to identify representative Bacteroides species	Forster et al.^63^	ftp://ftp.ebi.ac.uk/pub/databases/metagenomics/hgg_mags.tar.gz
External metagenomic sequencing data: The Inflammatory Bowel Disease Multi'omics Database	Proctor et al.^27^	SRA: PRJNA389280
External metagenomic sequencing data: The Human Microbiome Project	Loyd-Price et al. ^50^; Turnbaugh et al.^28^	SRA: PRJNA48479,SRA: PRJNA275349
External metagenomic sequencing data: MetaHIT: The European Union Project on Metagenomics of the Human Intestinal Tract	Qin et al.^29^	SRA: PRJEB2054
External metagenomic sequencing data: The 1000IBD project: multi-omics data of 1000 inflammatory bowel disease patients	Imhann et al.^30^	EGA: EGAS00001002702
External metagenomic sequencing data: “The treatment-naive microbiome in new-onset Crohn's disease”	Gevers et al.^31^	SRA: PRJNA237362
External metagenomic sequencing data: “Inflammation, Antibiotics, and Diet as Environmental Stressors of the Gut Microbiome in Pediatric Crohn’s Disease”	Lewis et al.^32^	SRA: SRP057027
Count tables, metadata, and the sequences of MAGs and viral regions analysis of IBDMDB	Nishiyama et al.^34^	ftp://ftp.genome.jp/pub/db/community/ibd-phage/

**Experimental models: Organisms/strains**		

Mouse C57BL/6J_SPF	Envigo, Israel	C57BL/6JOlaHsd
Mouse C57BL/6J_GF	Local breeding	C57BL/6NTac

**Oligonucleotides**		

B_Frag_upaY_F- CGCTCGGACAAAGAAGGACC	This study	N/A
B_Frag_upaY_R- ACTTCTACCCTACGACGACGA	This study	N/A
B_Frag_PSA_F- TGTGTAAATGATAGGAGGCTAGGG	This study	N/A
B_Frag_PSA_M- GGTGTTCCAAAAGACGAACGT	This study	N/A
rpsL_F-CCGAACTCTGCAATGCGTAA	Ben-Assa et al.^7^	N/A
rpsL_R -CGCGAACCAGTACGATTGAG	Ben-Assa et al.^7^	N/A
wcf_F-GGCCTCCTTCATCTCAGGTTTATCC	This study	N/A
wcf_R-GATAATCGCGGCACCCTATGGG	This study	N/A
mpi_F-AAGAGGGCTATGTGTTTCAGGACG	This study	N/A
mpi_R-CTGCGTGCGAGAGCTTCTTTG	This study	N/A

**Software and algorithms**		

Graphpad Prism	GraphPad Version 9.0 or higher	https://www.graphpad.com
FlowJo	Tree Star,Inc.	https://www.flowjo.com/
PhaseFinder v1.0	Jiang et al.^9^	https://github.com/XiaofangJ/PhaseFinder
MetaPhlan 4	Blanco-Miguez et al.^64^	https://github.com/biobakery/MetaPhlAn
gtdbtk v2.0.0	Chaumeil et al.^65^	https://github.com/Ecogenomics/GTDBTk
Bowtie2 v2.3.5.160	Langmead et al.^66^	http://bowtie-bio.sourceforge.net/bowtie2/index.shtml
NCBI Graphical Sequence Viewer v3.49.0	Rangwala et al.^67^	https://www.ncbi.nlm.nih.gov/projects/sviewer/
MicrobiomeAnalyst v2.0	Dhariwal et al.^68^	https://www.microbiomeanalyst.ca/
R v4.3.1	R Development Core Team	https://www.r-project.org/
RStudio 2023.06.1	R Studio Team	https://www.rstudio.com/
Phyloseq v1.44 (R package)	McMurdie et al.^69^	https://joey711.github.io/phyloseq/
Vegan v2.6-4 (R package)	Oksanen et al.^70^	https://CRAN.R-project.org/package=vegan
Maaslin2 v1.14.1 (R package)	Mallick et al.^71^	https://huttenhower.sph.harvard.edu/maaslin/
DeSeq2 v1.40.2 (R package)	Love et al.^72^	https://github.com/thelovelab/DESeq2
ggplot2 v3.4.3 (R package)	Wickham^73^	https://github.com/tidyverse/ggplot2
EnhancedVolcano v1.18.0 (R package)	Blighe et al.^74^	https://github.com/kevinblighe/EnhancedVolcano
Tidyverse v2.0.0 (R package)	Wickham et al.^75^	https://www.tidyverse.org/
ggpubr v0.6.0 (R package)	Kassambara^76^	https://rpkgs.datanovia.com/ggpubr/
rstatix v0.7.2 (R package)	N/A	https://github.com/kassambara/rstatix
ggtree v3.4.4 (R package)	Yu et al.^77^	https://github.com/YuLab-SMU/ggtree
BBDuk, part of the BBTools v37.50	Bushnell^78^	https://jgi.doe.gov/data-and-tools/bbtools/
Velvet v1.2.10	Zerbino et al.^79^	https://github.com/dzerbino/velvet
Velvet Optimizer v2.2.5)	N/A	https://github.com/tseemann/VelvetOptimiser
Prokka v1.12	Seemann^80^	https://github.com/tseemann/prokka
Proksee	Grant et al.^81^	https://proksee.ca/
MAFFT online service v7	Katoh et al.^82^	https://mafft.cbrc.jp/

### Resource availability

#### Lead contact

Further information and requests for resources and reagents should be directed to and will be fulfilled by the lead contact, Dr. Naama Geva-Zatorsky (naamagvz@gmail.com).

#### Materials availability

This study did not generate new unique reagents.

#### Data and code availability

• Data of metagenomic sequencing have been deposited at the SRA database under project accession number SRA: PRJNA779701. Bacteriophage Barc2635 genome has been deposited to the GenBank database under accession number GenBank: MN078104. Accession numbers for the existing, publicly available datasets analyzed in this study are listed in the key resources table.
• All software tools used in this paper are publicly available and are listed in the key resources table. This paper does not report original code, as no new code was generated.
• Any additional information required to reanalyze the data reported in this paper is available from the lead contact upon request.

### Experimental model and study participant details

#### Bacterial culture conditions

*B. fragilis* NCTC 9343 was grown in Brain heart infusion (BHIS) supplemented with 5 mg/L hemin (Alfa Aesar) in 1 N NaOH, and 2.5 μg/L vitamin K under strictly anaerobic conditions (80% N2, 10% H2, 10% CO2) at 37°C in an anaerobic chamber.

#### Animal experiments

All mouse work was in accordance with protocols approved by the local IACUC committee under approval numbers: IL-151-10-21 and IL-105-06-21.

4-5 weeks-old Germ-Free (GF) C57BL/6 mice (males and females) from the Technion colony were used. Mice were housed and maintained in a GF care facility and were provided with food and water *ad libitum*; they were exposed to a 12:12 h light-dark cycle at room temperature.

Humanized mice were generated by oral gavage of GF C57BL/6 mice with 200 μl mix of human fecal microbiota and cultured *B. fragilis* NCTC 9343. To prepare this mix, human feces from a single healthy human donor were suspended in sterile PBS 1:10 w/v (1 gram feces was suspended in 10 ml of sterile PBS). Next, 10^8^ CFUs of *B. fragilis* NCTC 9343 were resuspended in 1 ml of sterile PBS and added to 1 ml of fecal supernatant. *B. fragilis* NCTC 9343 were grown on Brain Heart Infusion agar plates (BHI, BD BBLTM) supplemented with 5 μg/ml hemin (Alfa Aesar) in 1 N NaOH and 2.5 μg/ml vitamin K (Thermo Fisher Scientific) in 100% EtOH, at 37°C in an anaerobic chamber, 85% N2, 10% CO2, 5% H2 (COY). The progeny of these mice were co-housed, randomized, and grouped before treatment.

Gnotobiotic mice monocolonized with *B. fragilis* were created by oral gavage of GF C57BL/6 mice with 200 μl of 5x10^8^ CFU\ml of *B. fragilis* NCTC 9343, grown in the same conditions as above. The mice were co-housed, randomized, and grouped before treatment. Both gavages, in humanized and monocolonized mice, were performed once.

For phage immunomodulation studies, GF mice were gavaged twice (on day 0 and on day 2) with *B. fragilis* NCTC 9343 (10^8^ CFU in 200 μl Bacteroides phage recovery medium (BPRM) media) and bacteriophage Barc2635 (10^9^ PFU in 200 μl 0.22-μm-filtered BPRM), *B. fragilis* only (10^8^ CFU in 200 μl BPRM media), or growth media as control. On day 10 mice were sacrificed for bacteria and immune phenotyping.

#### Human participants

Recruitment of IBD patients for this study was conducted at the Rambam Health Care Campus (RHCC). The study was approved by the local institutional review boards with study numbers 0052-17 and 0075-09 in which all patients consented to be included in it. Fecal samples were collected by the patients, prior to their clinic visit. The samples were then stored at -80°C until they were shipped to the laboratory for analysis.

In the cohort of 11 participants, Infliximab (HR) was administered to 4 patients, and Humira (HuR) was administered to 7 patients. The cohort included six males and five females. The median Body Mass Index (BMI) was 22.57, with a range of [16.94, 29.41]. The median age at the initiation of therapy was 36.9 years, ranging from [24.8, 58.4]. Of the participants, two were Israeli Arabs, and the remaining belonged to the Israeli Jewish population. Data regarding socioeconomic status was not documented.

Patient response classification was determined by a previously described decision algorithm.^83^^,^^84^ Briefly, clinical response was defined by the attending physician as clinical and/or endoscopic improvement of IBD-related symptoms coupled with a decision to continue therapy, at least 14 weeks after treatment initiation. Non-response was defined by lack of improvement or aggravation of clinical or endoscopic presentation or disease symptoms coupled with therapy change.

### Method details

#### Identification of DNA inversion regions

Representative Bacteroides species were selected from using the following approach: a database of human-associated microbial species was constructed from 118K metagenomics assembled genomes (MAGs) recovered from human-associated metagenomics samples acquired from Pasolli et al.,^61^ Almeida et al.,^62^ and Forster et al.^63^ Taxonomy assignment was performed using gtdbtk v2.0.0 and GTDB release 207 with the classify_wf program and default parameters.^65^ Overall, 34 *Bacteroides* species were identified of which 33 had species-level GTDB taxonomy assignments. For each of these species, the representative genome from GTDB was used for the PhaseFinder analysis. We have also included the genomes of three additional *B. fragilis* strains, *Phocaeicola dorei*, and *Parabacteroides distasonis*. Overall, 39 genomes were included. Information about the genomes is provided in Table S1.

PhaseFinder^9^ (v1.0) was used to identify invertible regions in metagenomics samples. The default parameters of PhaseFinder were used. Putative inversion regions were detected by identifying inverted repeats in Bacteroidales genomes using the ‘locate’ function. A database containing the inversion regions forward and reverse orientations was created using the tool’s ‘create’ function. Using the tool’s ‘ratio’ function, metagenomic samples from the publicly available cohorts were then aligned to the database, resulting in the ratio of reverse oriented reads out of all reads assigned to each region. We filtered the results by removing identified regions with <20 reads supporting either the forward or reverse orientations combined from the paired-end method, mean Pe_ratio <1% across all samples, and sites within coding regions of rRNA products. To compare DNA orientation patterns between CD and UC patients with those of healthy controls, we focused on sites that displayed a difference of over 10% between at least one of the groups. Each invertible region was manually curated to assess its coding regions, gene annotations, and their putative functions. Briefly, genomic regions were visualized online using the NCBI Graphical Sequence Viewer (version 3.47.0). Invertible regions with coding sequences (CDS) within them, were annotated according to the CDS name(s). Invertible regions lacking CDS within them were searched for CDSs that start in proximity to the invertible DNA sites (<200∼bp). CDSs in the region (four upstream and four downstream) were used to assess the functionality of the region. Regions containing or in proximity to rRNA and tRNA genes were filtered from the comparisons as well as invertible regions with no CDSs start in proximity to the inverted repeats.

#### Mice DSS-induced inflammation model

For DSS experiments, male and female mice were co-housed separately. Mice were randomized and grouped (water control and DSS separately) before treatment. Throughout the experiments, close attention was paid to monitor any distress responses exhibited by the mice: mice with a 20% decrease in body weight, immobility, or an extreme reaction to touch, were excluded from the experiments.

For acute DSS-induced colitis in humanized mice, the progeny of humanized mice were treated with 3% DSS in their drinking water for 9 days followed by 5 days of recovery before sacrificing for the experiment. The control animals were administered distilled water.

Fecal samples were obtained on days 0, 6, 9, and 14 (before DSS treatment, during, and after discontinuing DSS treatments), for further analyses.

For acute DSS-induced colitis in gnotobiotic mice colonized with *B. fragilis*, monocolonized mice were treated with 3% DSS in their drinking water for 7 days. The control animals were administered distilled water. Fecal samples were obtained on days 0 and 7 (before and after DSS treatment), for further analyses.

#### Mice inflammation measurement

Fecal supernatants were prepared after suspension in 1:10 sterile PBS and centrifugation at 4,500xg for 15 min. Enzyme-linked immunosorbent assay (ELISA) to measure calprotectin concentrations was performed using Mouse S100A8/S100A9 Heterodimer kit according to the manufacturer protocol [R&D systems]. Mice weights were assessed using an electronic scale at the same day-time.

#### Quantitative PCR for PSA promoter orientation

DNA was extracted from fecal samples (30-50 mg) using ZymoBIOMICS DNA Miniprep Kit [Zymo research]. The ‘ON’/’OFF’ orientation of the PSA promoter in the extracted DNA samples was determined by quantitative polymerase chain reaction (qPCR) using SYBR® Green mix [Thermo Fisher Scientific]. Two sets of primers were designed to target the PSA locus (Figures S1A–S1C). One set (B_Frag_upaY_F, B_Frag_upaY_R) targeted the reference gene *upaY*, the first gene immediately downstream to the promoter region, and used as a proxy to the number of bacteria in the samples. The second set of primers (B_Frag_PSA_F ,B_Frag_PSA_M) targeted the promoter region and would only produce a product when the orientation is ‘ON’. The ratio of ‘ON’/’OFF’ PSA orientation in the samples was calculated using the 2^−ΔΔCT^ method^85^ calculated against the PSA locked ‘ON’ results (100% ‘ON’ orientation).

*upaY* gene expression was determined by RT-qPCR with the *upaY* primers. RNA was extracted from fecal samples using zymoBIOMICS RNA miniprep kit [Zymo research]. Following DNAse treatment (NEB), reverse transcription of RNA to cDNA was performed using the qScript cDNA Synthesis Kit [Quantabio]. *upaY* mRNA levels were determined by qPCR using SYBR® Green mix [Thermo Fisher Scientific] with primers against *rpsL* as a reference gene (rpsL_F, rpsL_R). Controls for non-template and non-reverse transcription were included. Primer efficiency was determined for each set of primers.^86^ The 2^−ΔΔCT^ method was employed for the specificity fold change tests.

#### Mice feces sequencing

DNA was extracted from fecal samples (30-50 mg) using ZymoBIOMICS DNA Miniprep Kit [Zymo research]. Samples underwent quality control by Qubit fluorescence analysis to determine the concentration of DNA for downstream analysis (ThermoFisher, Cat. Q32850). Libraries were prepared using the Illumina Tagmentation DNA prep streamlined library preparation protocol according to the manufacturer’s instructions with a minimum of 50 ng of DNA starting mass and 8 cycles of PCR enrichment, ending with a fragment size of 550 bp. IDT for Illumina DNA/RNA UD indexes and Nextera DNA CD indexes were used (Illumina IDT, Cat. 20027213; Illumina Nextera, Cat. 20018708).

All libraries were diluted to 15 pM in 96-plex pools and validated on 100-cycle paired-ends read Miseq V2 runs (Illumina, Cat. MS-102-2002), before shipping to the US at 4 nM for sequencing on the Novaseq 6000 in S4 mode at 96-plex in a 300-cycle paired-end reads run, with an estimated read depth of 30 Gbp per sample (Illumina, Cat. 20028312). Final loading concentration of 600 pM. All sequencing runs were performed with a spike-in of 1% PhiX control library V3 (Illumina, Cat. FC-110-3001). The sequencing mean library size was 134,629,540.5 reads [range: 10,107,679 - 396,239,822].

#### Taxonomic profiling

Community profiling was performed using metaphlan4^64^ v4.0.059 with mpa database vJan21. For each sample, the forward reads were first aligned against the mpa database using bowtie2^66^ v2.3.5.160 (flags --**sam-no-hd --sam-no-sq --no-unal --very-sensitive**). Next, the resulting sam file was analyzed by metaphlan4 with default parameters, resulting in a merged relative abundances table.

#### Microbiome analysis

Initial visual exploration of sequenced data was conducted using the MicrobiomeAnalyst^68^ web platform and followed by a comprehensive statistical analysis using the Phyloseq^69^ version 1.44.0 and vegan^70^ version 2.6-4 packages in R v4.3.1. Read counts were transformed into relative abundances by normalization to the total number of reads per sample. Low-abundance filters were applied to discard species whose relative abundance did not reach 0.1% and did not appear in at least 20% of the samples. Alpha diversity (observed species and Shannon diversity) was calculated at the species level using the ‘plot_richness’ function. Beta diversity distance matrices (Aitchison distance) were ordinated using the ‘ordinate’ function and visualized using PCoA. Beta dispersion (Aitchison distance) was calculated using the vegan^70^ package’s ‘betadisper’ function. Plotting was performed using R packages tidyr version 1.3.0, reshape2 version 1.4.4, ggpubr version 0.6.0, ggplot2^73^ version 3.4.3, and EnhancedVolcano version 1.18.0.

#### Calprotectin in IBD patients’ fecal filtrates

Calprotectin levels were measured in each fecal sample using LIAISON calprotectin (catalog No. 318960) according to the manufacturer's instructions. The levels of calprotectin in the fecal samples were used as a measure of disease activity in IBD patients.

#### *In vitro* assays with fecal filtrates from IBD patients

*B. fragilis* NCTC 9343 was grown in Brain heart infusion (BHIS) supplemented with 5 mg/L hemin (Alfa Aesar) in 1 N NaOH, and 2.5 μg/L vitamin K to OD_600_∼0.6 and centrifuged at 4,500xg for 5 min. Bacterial pellets were washed twice with sterile PBS to remove BHIS components and then suspended with 1 mL supplemented M9 minimal media.

Patients’ fecal samples were suspended in sterile PBS (1:5), centrifuged at 4,500xg for 15 min, and supernatants were collected and filtered using the Medical Millex-VV Syringe Filter Unit, 0.22 μm, PVDF membrane.

For the *in vitro* assay, *B. fragilis* was cultured in a mixture of M9 and patients’ fecal supernatants in a 1:25:25 ratio (bacteria suspended in M9: M9: fecal supernatants) and grown at 37°C in an anaerobic chamber, 85% N2, 10% CO2, 5% H2 (COY). As a control, *B. fragilis* was grown in M9 and mixed with the same ratios but with sterile PBS instead of fecal filtrates. Subsequently, 200μl of each culture was collected for DNA extraction at OD_600_∼0.6 using ZymoBIOMICS DNA Miniprep Kit [Zymo research]. qPCR for PSA promoter orientation analysis was done using the primers used in mice experiments, with 3 technical replicates from each individual patient.

M9 Medium was prepared as follows: 

$$18.7\;\text{mM}\;{\text{NH}}_{4}\text{Cl}$$

$$42.2\;\text{mM}\;{\text{Na}}_{2}{\text{HPO}}_{4}$$

$$22\;\text{mM}\;{\text{KH}}_{2}{\text{PO}}_{4}$$

8.5mMNaCl

Supplements:

$$0.1\;\text{mM}\;{\text{CaCl}}_{2}.2H_{2}O$$

$$1\;\text{mM}\;{\text{MgSO}}_{4}.7H_{2}O$$

0.5%glucose

0.05%L-cysteine

5g/LHemin

$$2.5\;\text{mg}/\text{ml}\;{\text{VitK}}_{1}$$

$$2\;\text{mg}/\text{ml}\;{\text{FeSO}}_{4}.7H_{2}O$$

5ng/mlVitB12

#### Phage differential abundances from Nishiyama et al

Count tables, metadata, and the sequences of MAGs and viral regions from Nishiyama et al.^34^ analysis of the IBDMDB dataest were obtained from the following site:

ftp://ftp.genome.jp/pub/db/community/ibd-phage/.

Statistical analysis was done using the Phyloseq^69^ version 1.44.0 and DESeq2^72^ version 1.40.2 version 2.6-4 packages in R v4.3.1. The data was filtered to include only viral OTUs, and Low-abundance filters were applied to discard OTUs who had less than 10 read counts and did not appear in at least 20% of the samples. Read counts were normalized to relative abundances. Samples were split to low (<40%) or high (>60%) ratios of the PSA promoter ‘ON’ orientation, according to the PhaseFinder results. Differential abundance of viral OTUs between the groups were computed with R package DESeq2^72^ with default settings. Plotting was performed using R packages tidyr version 1.3.0, reshape2 version 1.4.4, ggpubr version 0.6.0, ggplot2 version 3.4.3, and EnhancedVolcano version 1.18.0. Phage-to-host abundance ratio was calculated for each phage-*B. fragilis* and phage-*B. thetaiotaomicron* pairs according to the bacterial host predictions done by Nishiyama et al.^34^

#### Bacteriophage Barc2635 isolation

Bacteriophage BARC2635 was isolated from raw sewage. Briefly, inflowing raw sewage for a waste-water treatment plant (WWTP) from Barcelona (Spain) was filtered through low protein binding 0.22 μm pore size polyethersulfone (PES) membrane filters (Millex- GP, Millipore, Bedford, Massachusetts) to remove bacteria. Isolated plaques were obtained by the double-agar layer technique.^87^ Briefly, tubes containing 2.5 ml of soft BPRM–agar kept at 45°C were inoculated with 1 ml of an exponential growth phase culture (OD600=0.3, corresponding to ca 2x10^8^ CFU / ml) of the host bacteria grown in BPRM broth and 1 ml of the filtered sewage sample. After gently mixing, the contents of each tube were poured onto a plate of BRPM-agar and incubated inside GasPak (BBL) jars at 37°C. Plaques were clearly spotted after 18 h of incubation.

For phage isolation, discrete well-isolated plaques were stabbed with a sterile needle and inoculated in a tube containing 5 ml of BRPM broth. Then 1 ml of a culture of *B. fragilis* NCTC 9343 in exponential growth was inoculated into the tube, which was then incubated for 18h at 37°C. After incubation, an aliquot of the culture was treated with chloroform (1:10 (v;v), vigorously mixed for 5 min and centrifuged at 16,000xg for 5 min.^88^ The supernatant containing the phage suspensions were further filtered through low protein binding 0.22 μm pore size polyethersulfone (PES) membrane filters (Millex-GP, Millipore, Bedford, Massachusetts), diluted and plated as indicated in the previous paragraph to verify the uniformity of the plaques. Then, one well differentiated plaque was stabbed and the whole operation was repeated to obtain a high titer, over 1x10^9^ plaque forming units (PFU), phage suspensions.

#### Phage genome sequencing

Phage particles were PEG-precipitated (6,000-12,000 MW, 8%), and isolated by a CsCl gradient; 33 g, 41 g, 55 g in 50 ml TM buffer (50mM Tris-Cl pH8.0, 10mM MgCl2), ultracentrifuged at average 152,000xg for 1.5hrs, and dialyzed overnight in 100mM Tris ph7.5, 1M NaCl, 1mM EDTA.^58^ Genomic DNA was extracted using phenol-chloroform as described.^89^

Illumina sequencing of Bacteroides phage Barc2635 was performed at the Biopolymers Facility, Harvard Medical School, Department of Genetics, producing paired-end reads of 150 bp. Adapter sequence removal and quality trimming was performed using BBDuk, part of the BBTools (v 37.50) suite of programs. The reads were further screened against NCBI’s UniVec_Core database (build 10.0) and the *B. fragilis* NCTC 9343 genome sequence using blastn and reads that returned a significant hit to either were removed. The phage genome was assembled de novo using Velvet 1.2.10 under a k-value determined by Velvet Optimizer (v. 2.2.5). The genome was annotated using a customized version of Prokka^80^ v1.12, altered to additionally utilize the profile Hidden Markov Model (HMM) libraries of Pfam^90^ version 35, TIGRFAM^91^ version 15, and the Clusters of Orthologous Genes^92^ (COGs, 2020 update, and PRotein K(c)lusters (PRK) portions of NCBI's Conserved Domain Database^93^ (refxx4) during annotation., submitted to NCBI, and assigned GenBank accession MN078104. Phage Genome map (Figure S3C) was visualized using the online tool Proksee (https://proksee.ca/, accessed on 21 January 2023).

#### Viral OTUs multiple alignment

Alignments and phylogenetic tree of whole genomes of viral OTUs identified as bacteriophages against *B. fragilis*^34^ as well as bacteriophage Barc2635 were generated using MAFFT^94^ online service (version 7). Multiple sequence alignment was done using the default settings of the site. An average linkage UPGMA guide tree was constructed using average-linkage UPGMA with the online MAFFT^94^ service and was visualized with R package ggtree^95^ (version 3.4.4).

#### Barc2635 susceptibility and competition assays

Frozen stocks of *B. fragilis* NCTC 9343 Δ*mpiM44*^43^ (genetically engineered bacteria that constitutively express PSA) and *B. fragilis* NCTC 9343 Δ*PSA*^44^ (lacks PSA biosynthesis locus) maintained in 25% glycerol at −80°C were thawed on Brain Heart Infusion agar plates (BHI, BD BBLTM) supplemented with 5 μg/ml hemin (Alfa Aesar) in 1 N NaOH and 2.5 μg/ml vitamin K (Thermo Fisher Scientific) in 100% EtOH, at 37°C in an anaerobic chamber, 85% N2, 10% CO2, 5% H2 (COY). Then strains were grown anaerobically for up to 3 days, and a single colony was picked for each bacterial strain, inoculated into 5 ml BPRM and grown anaerobically overnight to provide the starting culture for experiments. A dilution of 1:10 was done the following day and the diluted culture was incubated at the same conditions until mid-logarithmic phase of growth.

At the beginning of the experiment, comparable CFU amounts of each strain were verified at OD 0.5 (Δ*mpiM44* 2x10^8^ and Δ*PSA* 2x10^8^ ).

For susceptibility assays: 300μl of each strain was infected with Barc2635 in 1:100 ratio, and then incubated for 5 min in 37°C, followed by a centrifuge of 4,500g x 5 min and re-suspension with clean 300μl BPRM to get rid of free phages that did not infect the cells. The infected bacterial cells were added to 3 ml of the molten soft top agar and mixed well before being poured onto the bottom agar. The following day plaques were counted according to the formula:

PFU/ml = # of plaques / (dilution ^∗^ infection volume in ml).

For competition assays *in vitro*: Both bacterial strains (*ΔmpiM44* and Δ*PSA*) were grown at the same anaerobic conditions until mid-logarithmic phase of growth. Equal CFU’s were verified.

Next, bacterial cells were mixed in 1:1 ratio and then Barc2635 was added to the mix at 1:100 ratio. 200μl of Time point (Tp) 0 (starting point of the experiment) and Tp2 (2 h after Barc2635 infection) were taken to qPCR analysis.

*In vivo* competition was done by gavaging C57BL/6 mice with equal ratios of Δ*mpiM44* and Δ*psa*, waiting two days for the bacteria to settle in and then Barc2635 (10^9^ PFU in 200 μl 0.22-μm-filtered BPRM) was added on Tp0. 10 days after the inoculation mice fecal samples were collected for qPCR analysis. Primers detecting Δ*mpiM44* were designed to target the *wcf* gene of the PSA biosynthesis locus.

Primers detecting Δ*PSA* were designed to target the *mpi* gene.

#### Gut lamina propria preparation

For lamina propria immunophenotyping, mice colons were removed by cutting the colon from the cecum-colon junction to the anus. Fat tissue was carefully removed from colon tissue and further processed for single cell suspension preparation using lamina propria dissociation kit (Miltenyi), according to the manufacturer's protocol.

#### Flow cytometry

Cell preparations for flow cytometry analysis were performed in 5 ml tubes or U shape 96 wells plates. Single cells were washed with PBS and stained for live/dead staining using 1:1,000 in PBS, Zombie fixable viability dye (Biolegend) for 10 min, at room temperature, and washed once with FACS buffer, by centrifuge at 300xg for 5 min. For FcR blocking, cells were incubated with 0.5 μg CD16/CD32 antibody for 10 min on ice and proceeded to further staining without a washing step. Extracellular markers were stained with the relevant antibody panels for 30 min on ice and washed twice with FACS buffer, by centrifuge at 300xg for 5 min. After the last wash, cells were fixed with Foxp3 Fixation/Permeabilization working solution (Thermo) for 16 h at 4°C in the dark. For Intracellular staining, cells were permeabilized using 1X Foxp3 permeabilization buffer (Thermo) according to the manufacturer protocol. For intracellular blocking, 2 μl of 2% rat serum (Stemcell technologies) was added to each well for 15 min at room temperature and proceeded to further staining without a washing step.

To quantify the percentage of *B. fragilis* from monocolonized mice with PSA on their surface, feces from monocolonized mice with *B. fragilis* with or without phage were suspended 1:10 in ice cold PBS (mg/μl) and centrifuged at 300xg for 5 min, 4°C. Supernatants were separated from pellets and further centrifuged at 4,500xg for 5 min, 4°C. Bacterial pellets were resuspended in an ice cold FACS buffer, 1:10 from initial PBS suspension. 100 μl of resuspended bacteria were incubated with 1:1,000 rabbit antibodies (Antibodies preparation previously described; xx doi: 10.1128/iai.67.7.3525-3532.1999) against *B. fragilis* PSA for 30 min at 4°C. Bacteria were washed twice using an ice cold FACS buffer by centrifuge at 4,500xg for 5 min and then incubated with a donkey anti rabbit fluorophore conjugated secondary antibody. After staining steps, the bacteria were washed twice with an ice cold FACS buffer and finally resuspended in 500 μl ice cold PBS plus 1:1,000 Hoechst dye and analyzed by flow cytometry using FSC and SSC thresholds of 1,000, and logarithmic scale. Gating strategy is detailed in Figure S5. Antibody specificity for PSA was verified using PSA mutant *B. fragilis*. Non-specific binding controls were included.

### Quantification and statistical analysis

The specific sample sizes (n) for each experiment are provided in the figure legends, and definitions of statistical tests and p-values are presented in the figures or figure legends. The Wilcoxon rank sum test was employed to compare DNA invertible regions orientation patterns between CD and UC patients with those of healthy controls, and the Benjamini-Hochberg method was utilized to correct for multiple comparisons, with a false discovery rate (FDR) set at less than 0.1. For metagenomics samples, alpha diversity (observed species and Shannon diversity) was compared with the Kruskal–Wallis rank sum test. Beta diversity distance matrices (Aitchison distance) were compared using the Vegan^70^ package’s ‘adonis’ function (Permutational Multivariate Analysis of Variance). Differential abundance of species between mice groups and timepoints were computed with R package Maaslin2,^71^ version 1.14.1 with CLR transformation of the data and with an FDR (BH) set at less than 0.05. Differential abundances of viral OTUs abundances in Nishiyama et al.^34^ were computed with R package DESeq2^64^ with default settings and FDR (BH) set at less than 0.05.

## Supplemental information

Document S1. Figures S1–S5

Table S1. Phase variation in human datasets, related to Figure 1

Table S2. Phase variation in mouse model, related to Figure 2

Table S3. Title phage differential abundances in Nishiyama et al., related to Figure 3

Document S2. Article plus supplemental information
